# A Case of Strictured Œsophagus Terminating in Death by Starvation

**Published:** 1822-09

**Authors:** 


### Art. VII.—*A Case of Strictured Œsophagus terminating in Death by Starvation.* By Dr. Kinglake.

Mr. Thomas Jacobs, an eminent brewer of this town, aged forty-six, and of a sanguineous temperament, had been occasionally inconvenienced in swallowing, for upwards of a year and a half before he regarded it as an ailment deserving serious attention. During these last twelve months, the power of deglutition has been much impaired, and at times so obstructed as justly to excite considerable alarm. About six months were passed in gradually increasing difficulty of swallowing, until at length no substantial food could be forwarded to the stomach. It was arrested at a short distance above the cardiac entrance into the stomach, and, after being detained a few minutes in that situation, it was rejected by vomiting. For these last four months, it was almost uniformly found, on repeated and painful trial, to be impracticable to take sustenance in any other than a fluid form, and even that but slowly, scantily, and of no thicker consistency than milk. Neither the animal broths nor farinaceous infusions, if at all glutinous, could pass the obstructed part.

A probang, or bougie, had been repeatedly passed down to the strictured part by Sir Astley Cooper, and subsequently by Mr. Norman, of Bath; but, from the extreme narrowness of the aperture at the stricture, these instruments could have only come in contact with its upper portion, and could not have been forced through it. The area of the stricture, as it appeared on examination after death, would not admit of the passage of a common size probe; and the abridged cavity exhibited the aspect of having been occasioned by a disorganized and hardened structure, that would not have yielded to any pressure that could have been made on it consistently with human endurance, or even existence.

For twenty-eight days preceding death, not the smallest quantity of fluid seemed to pass into the stomach. Whatever was taken into the gullet was almost immediately thrown back, without either much effort or pain. At times an aching sensation, attended with indescribable distress, was experienced. No feeling of nausea, nor any thing like stomachic vomiting, ever occurred. The contractile action, by which the obstructed contents of the gullet above the strictured part were rejected, appeared to be solely exerted by the œsophagus. The stricture was constituted by altered structure, to the extent of three inches. The whole circumference of the diseased part was much enlarged and indurated, assuming a cartilaginous texture, the internal membranous surface of which was rugose and elevated into a cauliflower-like excrescence, studded with ulcerated points. The parts immediately above and below the stricture were highly vascular; in which appearance the whole interior of the stomach largely partook. The intestinal canal, with the exception of some small scybalous accumulations in different portions of its track, was empty. The abdominal and thoracic viscera were apparently sound. An adhesion of the right lobe of the lungs had taken place with the costal pleura, but without any aspect of pulmonary disease. The liver, and indeed all the viscera that were particularly examined, felt unusually dense and solid, which might have been induced by the long abstraction of nutriment that had existed. A sense of hunger, in the early stage of the obstruction, prevailed, but latterly it was not mentioned; that of thirst, however, was insatiably distressing.

Clysters, consisting of milk triturated with yolks of eggs, and mutton and beef broths, were often injected, which were sometimes retained for an hour or two, but was too commonly returned within that period. The skin was extensively sponged with milk every three or four hours during the last month, which was highly gratifying, and perhaps in some measure sustaining, to the patient. Quicksilver, to the extent of from one to two ounces, was given two or three times a-week; which, after occasioning a sense of great load at the strictured part, passed into the stomach and through the intestines, but rather in an unctuous than in a globular or metallic form. Globules were visible, but the greater part of the metal had been extinguished in the mucous and other secretions of the intestines, by the triturating influence of the peristaltic or transmitting action that had been exerted on it. No pain was occasioned by this process. The urinary secretion was not in natural quantity, but nearly so, and far exceeding any supply that could have been derived from liquids taken into the stomach. The declension of strength was very gradual; the emaciation was more rapid, and at length reached the utmost verge of both vital and material exhaustion. No instance, perhaps, ever occurred of more sheer starvation than this case presented, and that, too, from a mechanical rather than any other cause. The general condition of the viscera, with their respective functions, was natural and healthy; nor was there any state of systematic irritation that contributed to accelerate the extinction of life.

The probable origin of this stricture was in a morbidly irritable state of the mucous membrane of the œsophagus, which had been induced by a course of drinking fermented liquors,—if not intemperately, at least to an extent that must have had the noxious effect of deranging the healthy condition of the digestive organs, and that of the biliary, pancreatic, and other enteric secretions. This disordered state of the first passages was at length shown by violent retchings, occurring in the morning after any excess had been committed the preceding evening; which ultimately settled into an almost habitual daily vomiting. This retrogade, or inverted, action of the œsophagus had existed for some years, before much difficulty was experienced in deglutition. The part of the œsophagal tube in which the morbid irritability more particularly prevailed, finally became the seat of the destructive stricture. Membranous thickening, in certain temperamental liabilities to increasing distemper, is apt to proceed to ulceration, which, aided by the subsisting disordered excitement, may at once decompose or disorganize old vessels, and generate new ones, so as to alter and sink the primordeal structure into a confused mass of irremediable disease. From this wreck of natural fabric may arise various conditions of morbid texture, including even those of scirrhous or cancerous ulceration. In the instance under consideration, the constituent parts of the œsophagus, at the distempered part, had undergone changes from the healthy state, that exhibited a most aggravated and irretrievable condition of disease, strikingly assuming, if not actually possessing, the true cancerous character. The membranous, cellular, and muscular tissues were blended and lost in an indurated enlargement, resembling the texture of cartilage. Such morbid changes and accretions must have been of slow progress, but of too inveterate a nature to admit of any rational scheme of remedy. Medicinal influence could not reach such an unalterable establishment of disease; nor could any mechanical means, such as dilating the narrowed part with a probang or bougie, afford a chance of being beneficial. On the contrary, it would painfully exasperate the ailment, as was really the case in the instance recited, whenever it was attempted. In fact, no probang or bougie, however small in circumference, could have passed the very diminished, nay, almost obliterated, area of the strictured part; and the irritation attending the fruitless endeavour must, by inflicting hurtful violence, have occasioned great additional mischief.

Entire abstinence from substantial food, and exclusively taking mild nutritive fluids for sustenance, with counter-irritation, in the form of caustic, opposite the strictured part, would be most likely to prove serviceable, by giving the morbid excitement an opportunity of subsiding, and affording, by its removal, a possibility for any structural enlargements and indurations that may have formed to be decomposed and swept away by absorption. All morbid accessions of new parts, as well as those which are healthy, are generated and sustained by nutritive vessels. The stimulus of disease having produced them, will, whilst it endures, enable them to furnish the supply that may be necessary to increasing growth. If this stimulus be withheld, its diseased effects will cease; and thus distempers founded in altered structure may be finally overcome, and effectually cured.

If the contractile power situated in the muscular structure of the œsophagus, in common with that which exists in all the muscular and tubulated parts of the animal frame, be so irregularly or spasmodically excited as to induce an unequal and partial contraction of its cavity, without at all altering or involving the fabric of the part, it may very probably be relieved and cured by the influence of mechanical distention. This mode of remedy is effectual in urethral strictures from simple contraction, and may be also equally availing in similar affections of the œsophagus. It is important to discriminate between strictures in the œsophagus from altered structure, and those which arise from simple narrowing, without any organic affection of the contracted part. The former, generally speaking, are perhaps only the advanced or closing stage of the latter, which evinces the propriety of endeavouring to prevent, by the most efficient means, the one state from attaining to that of the other.

The afflicting starvation that literally and strictly terminated life in this case, is an appalling consideration for those who may be subjected to a similar disease. All the alleviation was rendered that circumstances would either allow or appeared to indicate. Inaccessible as the stomach and bowels were for nearly a month before death, even to all aqueous as well as nutritive liquids taken by the mouth, the dernier, and indeed only, resort lay in quicksilver; which, as before observed, slowly passed the bowels, and in some measure kept in action the peristaltic power, which, without such influence, would probably have been in a state of the utmost inertion. Opiate frictions over the strictured part, and on the region of the stomach, when severe pain was felt in these situations, were employed with temporary relief. Sponging the cutaneous surface with tepid milk, at short intervals, was gratifying and apparently sustaining.

The action of the heart and arteries was steady and moderately firm until within a few days of death, when it became gradually more and more enfeebled, till it was hardly discoverable at the wrist. An irksome restlessness, with an importunate desire for frequent change of position, and an undiminished avidity for taking liquids into the mouth, and passing them on to the strictured part, from whence they were speedily returned, marked the closing scene of life.

The temperature, which had been natural until the heart began to lose its contractile force, then became deficient on the general surface, as well as on the extremities. The vision also, about the same time, was obscured, and the hearing dull; but the skin acquired an increased or distempered sensibility, so as to cause the patient to exclaim, if either the bed-clothes were wrinkled on the feet, or if the hands were slightly pressed. Intervals of tranquil and refreshing sleep were experienced, and seemed to have been shortened only by the awakening urgency of insupportable thirst. Life reached its final exhaustion without any apparent struggle: the act of dying, indeed, had the aspect of renewed sleep, and its consummation appeared to be equally calm and unruffled, which occurred on the 13th instant.

*Taunton; July 20th,* 1822.

