# Erratum in the Last Number

**Published:** 1822-10

**Authors:** 


ERRATUM *in the last Number.*

Dr. KINGLAKE's Paper, page 214, line 22, for *was too* read *were.*

